# The healthcare and economic burden associated with inadequate risk factor control for type 2 diabetes in Hong Kong: A population‐based modelling study

**DOI:** 10.1111/dom.70081

**Published:** 2025-09-10

**Authors:** Aidi Liu, Yumeng Shao, Jiayin Chen, Carmen S. Ng, Yanyan Wu, Xuechen Xiong, Cindy L. K. Lam, Eric Y. F. Wan, Jianchao Quan

**Affiliations:** ^1^ School of Public Health, LKS Faculty of Medicine The University of Hong Kong Hong Kong SAR China; ^2^ School of Public Health University of Pittsburgh Pittsburgh Pennsylvania USA; ^3^ School of Population and Global Health McGill University Montreal Quebec Canada; ^4^ Boehringer Ingelheim International GmbH New York USA; ^5^ Department of Family Medicine and Primary Care, LKS Faculty of Medicine The University of Hong Kong Hong Kong SAR China; ^6^ Department of Pharmacy and Pharmacology, LKS Faculty of Medicine The University of Hong Kong Hong Kong SAR China; ^7^ Laboratory of Data Discovery for Health Hong Kong Science Park Hong Kong SAR China

**Keywords:** glycaemic control, health economics, population health, public health, risk factor control, type 2 diabetes

## Abstract

**Aim:**

To estimate the healthcare and economic burden associated with improved risk factor control for people with type 2 diabetes in Hong Kong over 10 years.

**Materials and Methods:**

We obtained population‐based data from electronic healthcare records of the Hong Kong Hospital Authority. Risk factor targets were defined by American Diabetes Association guidelines. We applied a validated patient‐level diabetes outcomes model (Chinese Hong Kong Integrated Modelling and Evaluation) to estimate the health and economic outcomes for all individuals with type 2 diabetes (*n* = 526 672) in Hong Kong in 2021. Immediate risk factor control was compared to baseline over 10 years. Costs were estimated from a healthcare provider perspective.

**Results:**

Most people (84.9%) failed to achieve optimal combined risk factors control (glycated haemoglobin, blood pressure and low‐density lipoprotein‐cholesterol) at baseline. Combined control was associated with population‐level increases in quality‐adjusted life‐years (QALYs) of 17 605 and healthcare cost savings of US$ 106.7 million over 10 years. Glycaemic control solely yielded the greatest QALY increases and had the highest cost savings (US$ 29.0 million) over 10 years.

**Conclusions:**

The substantial population health and economic burden of inadequate risk factor control for individuals with diabetes in Hong Kong can potentially be mitigated through enhanced adherence, highlighting the need for effective and intensive interventions.

## 1 INTRODUCTION

The global diabetes epidemic is a pressing public health concern, with the prevalence rising at an alarming rate. According to the International Diabetes Federation (IDF), approximately 588.7 million adults were living with diabetes in 2024, with this number projected to reach 852.5 million by 2050.^1^ Diabetes and its related complications have now become a major worldwide public health issue that will affect almost all populations in both developed and developing countries.

In Hong Kong and elsewhere, diabetes is a pervasive and debilitating chronic disease that poses a significant burden on the healthcare system and is a leading contributor to mortality. The prevalence of prediabetes in Hong Kong is 8.9% while the prevalence of type 2 diabetes is 10.3%.^2^ The risk of type 2 diabetes increases significantly with age, rising from 0.6% in individuals aged 15–24 years to 19.0% in those aged 65–84 years.^3^

The economic and clinical burden of diabetes is substantial, with significant healthcare expenditures attributed to managing and treating diabetes and related complications. In China, the projected total cost of diabetes is expected to reach US$ 355.2 billion in 2025.^4^ Treating complications accounts for most (84.6%) of diabetes‐related healthcare expenditures, with only 8.1% and 6.7% spent on oral medication and insulin costs.^5^ Effective management of major risk factors, including glucose levels, blood pressure (BP) and lipids, is crucial in reducing the risk of mortality and diabetes‐related complications. However, only 50.1% of individuals on hyperglycaemic treatment achieved adequate glycaemia control, leading to increased health and economic burden.^6^ In Hong Kong, individuals with diabetes have an average annual healthcare expenditure approximately 1.6 times higher than those without diabetes, resulting in an incremental cost of US$ 3739 per person. Diabetes‐related medical expenditure was estimated at HK$ 18.6 billion in 2023, accounting for 6.5% of the total medical expenditure.^7^, ^8^

The Coronavirus Disease 2019 pandemic further disrupted diabetes care and impacted the management and monitoring of the disease.^9^ Delays in treatment and disruptions in care processes have led to worse health outcomes and higher healthcare costs.^10^ In Hong Kong, hospitalisation rates for severe hyperglycaemia and hypoglycaemia among people with diabetes decreased by over 20% during the initial outbreak period, compared to both the previous year and the preceding months.^11^ Reductions in acute hospitalisations may reflect glycaemic, BP and lipid management such as reduced access or delayed presentation. With regular outpatient services reduced as resources were diverted to acute healthcare services, people with chronic conditions were likely to experience suboptimal risk factor control periods. Disruptions in risk factor control may contribute to adverse health outcomes over time. Therefore, to inform public health policy, it is essential to estimate the potentially avoidable clinical and economic burden from inadequate control of risk factors among people with type 2 diabetes in Hong Kong.

## 2 METHODS

### 2.1 Data sources

Hong Kong has a population of 7.5 million (92% Chinese). Universal public health care in Hong Kong is provided by the Hospital Authority, a statutory body that manages public hospitals and ambulatory clinics, and provides care for 95% of people with diabetes in Hong Kong.^12^ The Clinical Management System (CMS) is the Hospital Authority's health informatics system, linking health records via unique patient identity numbers, including demographics, deaths, admissions, attendances, laboratory tests, diabetes‐related complications and prescribed medications. Clinical outcomes were extracted from the Hospital Authority CMS dataset using diagnostic codes specified in the Chinese Hong Kong Integrated Modelling and Evaluation (CHIME) model.^13^

### 2.2 Study population

We included adults diagnosed with type 2 diabetes from January to December 2021. We defined type 2 diabetes according to the diagnosis code for diabetes in the International Classification of Disease (ICD‐9). Individuals with a diagnosis code for type 1 diabetes were excluded.

### 2.3 Model development and updating

In this study, we employed the updated CHIME simulation model, a validated outcomes model for diabetes and related complications in Chinese (East Asian) populations.^13^ The CHIME model was updated using data from a population‐based cohort of 96 360 individuals diagnosed with type 2 diabetes in Hong Kong between 2013 and 2017, with follow‐up to April 2022 (mean follow‐up: 5.9 years).^13^, ^14^ This dataset was used to refit models for mortality, diabetes‐related complications and time‐varying progression of biomarkers. Haemodialysis was added as a new model outcome. The updated CHIME model included 13 risk equations to predict mortality, haemodialysis and major microvascular and macrovascular complications (including myocardial infarction, ischaemic heart disease, heart failure, cerebrovascular disease, peripheral vascular disease, neuropathy, lower limb amputation, skin ulcer, chronic kidney disease, retinopathy and cataracts), as well as an updated biomarker progression model incorporating body mass index (BMI), glycated haemoglobin (HbA1c), systolic BP and low‐density lipoprotein (LDL) cholesterol. For time‐to‐event outcomes, various parametric models (exponential, Weibull, log‐logistic, log‐normal, Gompertz and logistic) were tested and selected based on the Akaike Information Criterion (AIC). Risk equations were derived using parametric proportional hazard models (except for mortality, which used logistic regression, and retinopathy, which used a flexible parametric survival model). Biomarker progression models were developed using linear and logistic regression (see details in Appendix 1, Supporting Information S1). The CHIME model was developed and validated using the Hospital Authority's Hong Kong population data, and further details and modifications have been previously described.^13^

### 2.4 Modelling risk factor scenarios

Risk factor control scenarios were simulated for the cohort of people diagnosed with type 2 diabetes in Hong Kong in 2021, using the updated CHIME model to project clinical and economic outcomes over a 10‐year period. Outcomes were simulated at both 5‐ and 10‐year time points. Risk factor control was defined by Hong Kong reference framework and American Diabetes Association (ADA) guidelines (Table S1) on HbA1c targets <7% (53 mmol/mol), BP <130/80 mmHg and LDL‐cholesterol <2.6 mmol/L (<1.8 mmol/L for pre‐existing cardiovascular diseases). Four scenarios were simulated: individual control of glycaemia, BP and cholesterol; and multifactorial (glycaemia, BP and cholesterol). A 10‐year time horizon was chosen to capture the long‐term clinical and economic consequences of inadequate control of risk factors, allowing for disease progression and complications that may take years to manifest. The impact of inadequate risk factor control was evaluated by comparing immediate control, where the risk factors were assumed to reach their targets in the first year and continuously meet targets for the following 10 years, to the biomarker progression model in the updated CHIME which has no control of the risk factors at all.

### 2.5 Statistical analysis

The modelling outcomes included cumulative incidence and time to onset of complications, time‐paths risk factors, life expectancy, quality‐adjusted life years and direct costs arising from the treatment of diabetes‐related complications. Progression of other physiological variables (i.e., excluding BMI, HbA1c, systolic BP and LDL‐cholesterol) was assumed to constantly meet the target over the duration of the analyses to evaluate the impact of the risk factors of interest. Missing data for biomarkers at baseline was filled using sex‐specific sample means.

Quality‐adjusted life‐years (QALYs) were calculated by taking the baseline utility value for type 2 diabetes without complications and adding disutility for each occurring condition to create annual utility values for each individual. Utility values for diabetes with and without complications were taken from a meta‐analysis of Asian participants.^15^

The annual direct healthcare costs associated with diabetes‐related complications in the year of event and subsequent years in Hong Kong were derived from published sources.^16^ Direct healthcare costs of managing diabetes and related complications were calculated from a healthcare provider perspective and inflated to 2024 US dollars (US$) using the implicit price deflator of gross domestic product (GDP) published by the Census and Statistics Department. We applied a discount rate of 3% for costs and benefits.

### 2.6 Sensitivity Analysis

To assess the robustness of our findings, we conducted three sensitivity analyses: (1) varying the time to achieve risk factor targets (from 1 year to 3 and 5 years); (2) assuming partial achievement of control among the population (from 100% to 50% and 75%); and (3) adjusting the utility values (higher and lower bounds).

### 2.7 Ethics approval

This study was approved by the Institutional Review Board of the University of Hong Kong Hospital Authority Hong Kong West Cluster (ref.: UW 21‐297).

## 3 RESULTS

### 3.1 Baseline characteristics

The study population consisted of 526 672 people with type 2 diabetes in 2021 (50.3% female; Table S2). The study sample had an average age of 68.2 years, an average diabetes duration of 6.7 years and an average BMI of 25.8 kg/m^2^.

Most (*n* = 447 310; 84.9%; Table 1) did not achieve optimal targets for the three risk factors combined (HbA1c, BP and LDL‐cholesterol) at baseline: 250 685 (47.6%) people had HbA1c ≥7% (53 mmol/mol); 359 968 (68.3%) had BP ≥130/80 mmHg; and 99 307 (18.9%) had LDL‐cholesterol ≥2.6 mmol/L.

**TABLE 1:** People did not meet the target of risk factor control at baseline.

	Number of people did not meet target (*N*)	Percentage (%)	Number of males did not meet target (*N*)	Percentage (%)	Number of females did not meet target (*N*)	Percentage (%)
HbA1c	250 685	47.6%	126 931	24.1%	123 754	23.5%
BP	359 968	68.3%	181 364	34.4%	178 604	33.9%
LDL	99 307	18.9%	45 838	8.7%	53 469	10.2%
HbA1c + BP + LDL	447 310	84.9%	222 991	42.3%	224 319	42.6%

Abbreviations: BP, solely control of blood pressure; HbA1c, solely control of glycated haemoglobin; HbA1c + BP + LDL, combined control of HbA1c, blood pressure and LDL‐cholesterol; LDL, solely control of LDL‐cholesterol.

### 3.2 Death rate

Death rates comparing baseline (no risk factor control) with each risk factor control scenario are presented in Table S3. While mortality rates among individuals with controlled risk factors initially exceeded baseline rates, longitudinal analysis revealed lower mortality rates over longer time periods.

### 3.3 QALY gains

Immediate control of blood glucose, BP and LDL‐cholesterol was associated with population‐level QALY gains of 17 605 and 31 905 at 5 and 10 years, respectively, compared with the simulated risk factors time‐path progression (Table 2). When analysing control of individual risk factors, glycaemic control was associated with the greatest QALY gains at the population level, with 31 165 QALYs gained over 10 years compared to control of BP (3033 QALYs) and LDL‐cholesterol control (177 QALYs).

**TABLE 2:** Population‐level health and economic outcomes associated with risk factor control.

	All		Male		Female	
	5‐year	10‐year	5‐year	10‐year	5‐year	10‐year
Quality‐adjusted life years						
HbA1c	17 737	31 165	10 832	19 683	6905	11 482
BP	1756	3033	767	671	989	2362
LDL	276	177	169	−262	107	439
HbA1c + BP + LDL	17 605	31 905	11 515	20 380	6090	11 525
Life years gained						
HbA1c	20 017	35 136	12 260	22 253	7757	12 883
BP	1917	3318	929	818	988	2500
LDL	365	255	235	−311	130	566
HbA1c + BP + LDL	19 610	35 252	13 049	23 047	6561	12 205
Cost savings, US$						
HbA1c	3.4 m	29.0 m	1.1 m	12.6 m	2.3 m	16.4 m
BP	4.0 m	11.5 m	−8.6 m	−12.3 m	12.5 m	23.8 m
LDL	−3.5 m	4.5 m	−3.6 m	7.2 m	0.2 m	−2.6 m
HbA1c + BP + LDL	32.4 m	106.7 m	0.4 m	10.7 m	31.9 m	95.9 m

*Note*: This table presents population‐level changes in QALY gains, life expectancy gains and cost savings under four different risk factor control scenarios: sole control of glycated haemoglobin, blood pressure, LDL‐cholesterol and combined control of all three risk factors. Results are shown by time frame (5 and 10 years) and by gender (male and female). Combined control of HbA1c, blood pressure and LDL‐cholesterol resulted in the greatest QALY gains and cost savings over a 10‐year period. Among single risk factor control strategies, HbA1c control achieved better outcomes than either blood pressure control or LDL‐cholesterol control. Negative cost savings indicate an increase in costs compared to the reference scenario.

Abbreviations: BP, solely control of blood pressure; HbA1c, solely control of glycated haemoglobin; HbA1c + BP + LDL, combined control of HbA1c, blood pressure and LDL‐cholesterol; LDL, solely control of LDL‐cholesterol; m, million.

We further analysed the composition of QALY gains from the reduction of diabetes, excess weight and 10 diabetes‐related complications (amputation, skin ulcer, myocardial infarction, stroke, ischaemic heart disease, heart failure, peripheral vascular disease, neuropathy, retinopathy and haemodialysis). For the combined risk factor control (HbA1c, BP and LDL‐cholesterol), most of the QALY gains are from the prevention of stroke, retinopathy, heart failure and haemodialysis. For individual risk factor control, the QALYs are gained from different reasons: prevention of retinopathy for glycaemic control, stroke for BP control and haemodialysis for LDL‐cholesterol control.

### 3.4 Healthcare savings

Control of all three risk factors was also associated with healthcare cost savings of US$ 32.4 and 106.7 million over 5 and 10 years. At 5 years, BP control had the highest projected healthcare cost savings with US$ 4.0 million, followed by glycaemic control (3.4 million). At 10 years, glycaemic control has the highest cost savings of US$ 29.0 million, followed by BP control (11.5 million) and LDL‐cholesterol control (4.5 million). Discounted results are shown in Table 2. In scenarios where cost savings were negative, this represents a net increase in healthcare costs relative to the reference group.

**FIGURE 1:**
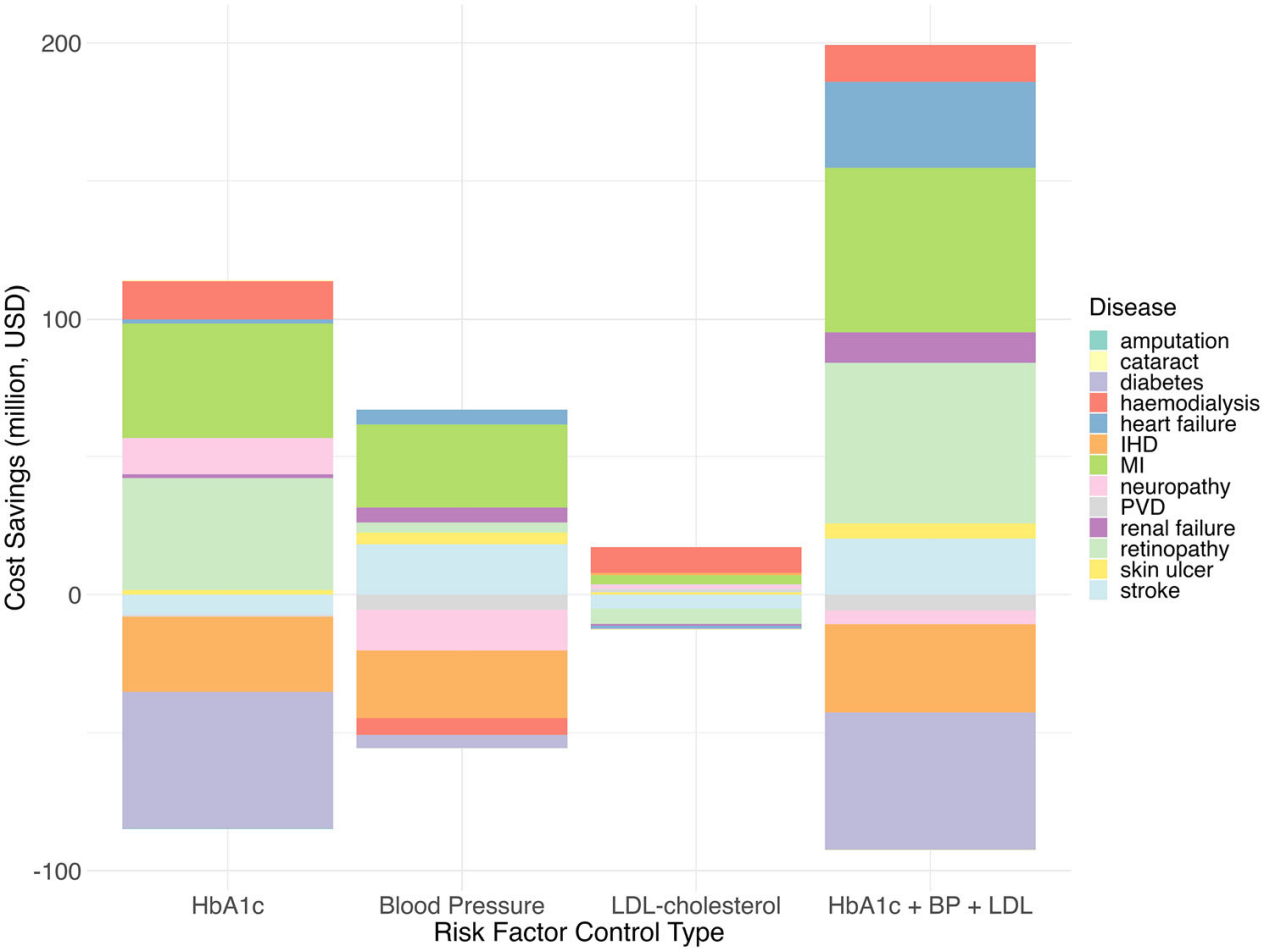
Ten‐year cost savings (in million USD) associated with improved solely control of glycated haemoglobin (HbA1c), solely control of blood pressure (Blood Pressure), solely control of low‐density lipoprotein‐cholesterol (LDL‐cholesterol) and combined risk factor control among people diagnosed with type 2 diabetes in Hong Kong in 2021. Stacked bars represent the cumulative cost savings from major diabetes‐related complications at 10 years, as estimated by the Chinese Hong Kong Integrated Modelling and Evaluation simulation model. Each colour represents a specific complication, including amputation, cataract, skin ulcer, myocardial infarction (MI), ischaemic heart disease (IHD), heart failure, peripheral vascular disease (PVD), renal failure, neuropathy, retinopathy, haemodialysis, stroke and diabetes progression. Combined control of glycated haemoglobin, blood pressure and LDL‐cholesterol yields the greatest total cost savings, mainly due to reductions in both cardiovascular and microvascular complications. Negative cost savings indicate an increase in costs compared to the reference scenario. HbA1c + BP + LDL, combined control of HbA1c, blood pressure and LDL‐cholesterol.

For each risk factor, we analysed the composition of the healthcare savings by diabetes and related complications (amputation, cataract, skin ulcer, myocardial infarction, stroke, ischaemic heart disease, heart failure, peripheral vascular disease, chronic kidney disease, neuropathy and haemodialysis; Figure 1). Among the 12 diabetes‐related complications, heart failure and retinopathy yielded the greatest cost savings primarily due to a significant decline in newly diagnosed cases. Controlling risk factors resulted in cost savings related to myocardial infarction in all scenarios.

Combined control of blood glucose, BP and LDL‐cholesterol yielded greater cost savings compared with controlling each risk factor separately. Most cost savings were attributed to myocardial infarction, retinopathy and heart failure. Notably, retinopathy accounted for the largest proportion of cost savings with US$ 30.1 million at 5 years, followed by myocardial infarction and heart failure, while myocardial infarction yielded the most cost savings of US$ 59.7 million at 10 years, followed by retinopathy and heart failure. The savings from heart failure and myocardial infarction were primarily driven by the high treatment costs associated with these conditions.

### 3.5 Individual‐level changes

Individual‐level improvements in QALYs and healthcare savings were relatively modest (Table 3). Combined control of all three target risk factors resulted in per‐person QALY gains of 0.0396 over 5 years and 0.0713 over 10 years, along with healthcare cost savings of US$ 72.4 and US$ 238.5, respectively.

**TABLE 3:** Individual‐level health and economic outcomes associated with risk factor control.

	All		Male		Female	
	5‐year	10‐year	5‐year	10‐year	5‐year	10‐year
Quality‐adjusted life years						
HbA1c	0.0708	0.1243	0.0853	0.1551	0.0558	0.0928
BP	0.0049	0.0084	0.0042	0.0037	0.0055	0.0132
LDL	0.0028	0.0018	0.0037	−0.0057	0.0020	0.0082
HbA1c + BP + LDL	0.0396	0.0713	0.0516	0.0914	0.0271	0.0514
Life years gained						
HbA1c	0.0798	0.1402	0.0966	0.1753	0.0627	0.1041
BP	0.0053	0.0092	0.0051	0.0045	0.0055	0.0140
LDL	0.0037	0.0026	0.0051	−0.0068	0.0024	0.0106
HbA1c + BP + LDL	0.0438	0.0788	0.0585	0.1034	0.0292	0.0544
Cost savings, US$						
HbA1c	13.53	115.68	8.68	99.23	18.52	132.55
BP	10.94	31.95	−47.22	−67.79	70.00	133.23
LDL	−34.81	45.10	−78.83	155.24	2.93	−49.32
HbA1c + BP + LDL	72.35	238.47	1.89	48.20	142.38	427.61

*Note*: This table presents individual‐level changes in quality‐adjusted life‐years gains, life expectancy gains and cost savings under four different risk factor control scenarios: sole control of glycated haemoglobin, blood pressure, LDL‐cholesterol and combined control of all three risk factors. Results are shown by time frame (5 and 10 years) and by gender (male and female). Negative cost savings indicate an increase in costs compared to the reference scenario.

Abbreviations: BP, solely control of blood pressure; HbA1c, solely control of glycated haemoglobin; LDL, solely control of LDL‐cholesterol.

All risk factor control methods led to QALY gains over 5–10 years. For single risk factor control, glycaemic control yielded the greatest cost savings (US$ 13.5 and US$ 115.7 over 5 and 10 years). Controlling LDL‐cholesterol alone only yielded savings over the longer term.

### 3.6 Analysis by sex

Glycaemic control was associated with the largest QALY gains for both men and women. LDL‐cholesterol control had a notably more beneficial effect for women than men.

**FIGURE 2:**
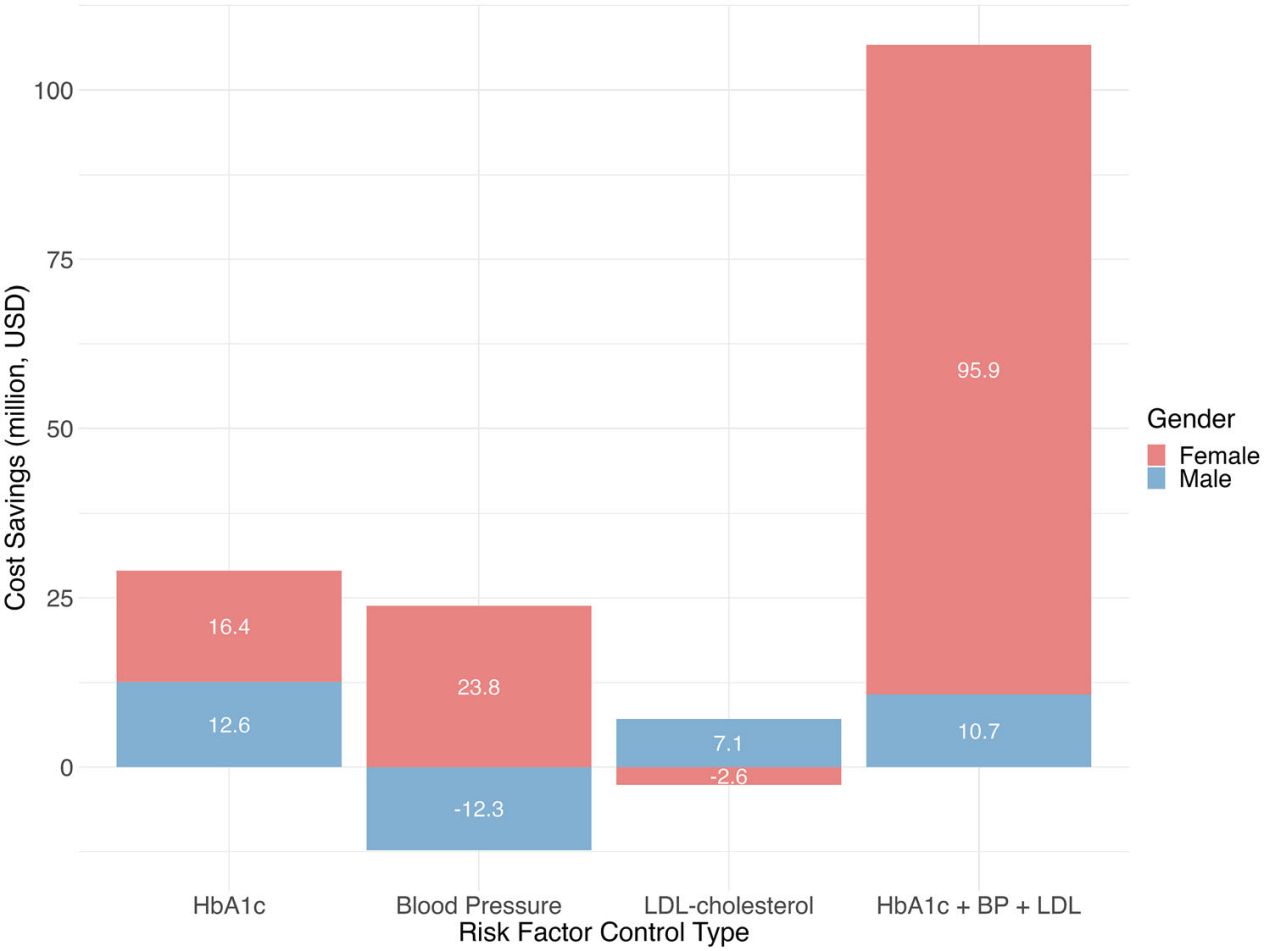
Ten‐year projected cost savings (in million USD) by gender for different risk factor control strategies in people diagnosed with type 2 diabetes in Hong Kong in 2021. Stacked bars represent the 10‐year cumulative cost savings (in million US dollars) from improved solely control of glycated haemoglobin (HbA1c), Blood Pressure (solely control of blood pressure), solely control of low‐density lipoprotein‐cholesterol (LDL‐cholesterol) and combined risk factor control among people diagnosed with type 2 diabetes in 2021, stratified by gender. Red and blue segments represent women and men respectively. The effect of different risk factor control strategies varied markedly by gender. For women, combined control of glycaemia, blood pressure and LDL‐cholesterol resulted in cost savings that were more than nine times higher than those for men. In contrast, for men, sole control of HbA1c led to the greatest cost savings among all strategies. These findings suggest gender‐specific differences in health economic benefits from various diabetes management approaches. Negative cost savings indicate an increase in costs compared to the reference scenario. HbA1c + BP + LDL, combined control of HbA1c, blood pressure and LDL‐cholesterol.

For cost savings, men and women exhibited distinct patterns across risk factor control scenarios (Figure 2). Among men, glycaemic control produced the largest cost savings, amounting to US$ 1.1 million over 5 years and US$ 12.6 million over 10 years. LDL‐cholesterol control resulted in increased costs over 5 years but shifted to cost savings at 10 years (US$ 7.1 million). In contrast, BP control was associated with increased costs at both 5 and 10 years (–US$ 12.3 million at 10 years). Combined control of all three risk factors resulted in additional cost savings for men (US$ 10.7 million over 10 years). Among women, BP control yielded the greatest cost savings, with US$ 12.5 million saved over 5 years and US$ 23.8 million over 10 years. Glycaemic control also resulted in substantial savings (US$ 16.4 million over 10 years). All control methods resulted in cost savings for women at 10 years except that LDL‐cholesterol control was associated with increased costs (–US$ 2.6 million). We noticed that combined control of all three risk factors led to the largest cost savings for both sexes, but the benefit was much more pronounced in women (US$ 95.9 million over 10 years), which was almost nine times greater than that observed in men (US$ 10.7 million).

### 3.7 Sensitivity analysis

As expected, for the partial control scenarios, increasing the proportion of the population achieving risk factor control led to greater QALY gains and cost savings across all strategies. Among single risk factor strategies, HbA1c control consistently provided the greatest QALY gains, while combined control yielded the highest cost savings, which is consistent with the main findings of our study. In the time‐varying to target scenarios, lower progress to achieve risk factor control targets generally resulted in reduced QALY gains and cost savings. When varying the utility values, the main trends for most interventions remained consistent with the primary analysis, with greater QALY gains over 10 years than over 5 years. Details of sensitivity analysis are provided in Tables S4 and S5.

## 4 DISCUSSION

This large cohort modelling study found that achieving and maintaining optimal risk factor control in people with type 2 diabetes is associated with reductions in health and economic burdens at the population level in Hong Kong. We found that healthcare cost savings associated with risk factor control were evident at 5 years, with greater benefits accruing over a 10‐year period. Our findings also indicate that joint control of all three factors leads to superior outcomes in healthcare savings compared with isolated control strategies. Improvements in health and economic outcomes result from the reduced incidence of diabetes‐related complications and longer life expectancy associated with improved risk factor control. Our risk factor targets were based on guidelines from the ADA (United States) and Health Bureau (Hong Kong), which are similar to those in China, Singapore and the UK.^17^, ^18^, ^19^, ^20^, ^21^, ^22^

The substantial health and economic impacts of risk factor control, such as those from poor glycaemic control, have been shown elsewhere.^6^, ^23^ We found fewer patients met their BP control targets at baseline than for glycaemia or LDL‐cholesterol. Our analysis further demonstrated that simultaneous control of HbA1c, BP and LDL‐cholesterol resulted in both improved QALYs and reduced healthcare costs. The QALY gains in the combined control strategy were primarily attributed to three mechanisms: prevention of retinopathy through glycaemic control, reduction in stroke incidence through BP management and decreased heart failure events through the synergistic effects of controlling all three risk factors (glycaemia, BP and LDL‐cholesterol) (Figure S1). This finding aligns with previous studies emphasising the importance of comprehensive cardiovascular risk factor management in diabetes.

When focusing on isolated risk factor control, we found solely glycaemic control contributed greater QALY gains than BP and LDL‐cholesterol. Accordingly, we found glycaemic control had the greatest projected healthcare cost savings for 5 years, nearly 1.75 times greater than that of BP control, and not to mention solely LDL‐cholesterol is not cost saving. The difference in the effect of cost savings between glycaemic control and other risk factors was increasing with a longer time period. Additional healthcare savings could be justifiable considering the projected population‐level cost savings when risk factor control is achieved, in addition to the reduced health burden. Given the cost savings, this could be particularly aimed at glycaemic and BP control in a shorter time period, such as anti‐hypertensives, glucose optimisation and additional monitoring, rather than LDL‐cholesterol. The factors that can cause poor risk factor control, such as patient compliance and self‐management, warrant particular attention and further research. This is especially important given we found the benefit at the individual level to be small, despite the considerable population gains, as described by Geoffrey Rose ‘Prevention Paradox.’^24^

Our study identified significant sex‐based differences in risk factor control outcomes. Women tended to derive greater QALY gains from isolated glycaemic control in the short term, while men gained more from a combined control strategy, which may reflect differences in cardiovascular risk profiles and health behaviours. Analysis of healthcare cost savings from concurrent control of glycaemic levels, BP and LDL‐cholesterol revealed marked gender differences, with women accounting for approximately 90% of total cost reductions. Among male participants, glycaemic control alone yielded the highest cost savings, while isolated BP management resulted in increased costs. These gender‐specific variations in healthcare costs may be attributable to differences not fully captured by the simulation model's parameters. While our model included key behavioural factors such as smoking and alcohol consumption, other uncaptured aspects—such as differences in healthcare utilisation, medication adherence, self‐management of lifestyle, social support or undiagnosed comorbidities—may also contribute to the observed differences in outcomes between sexes. Several mechanisms may explain the greater health and economic gains in women. Women with type 2 diabetes typically present with a higher burden of modifiable risk factors and have a higher relative risk of cardiovascular complications and mortality compared to men.^25^ Women are also less likely to receive guideline‐recommended therapies, so improved risk factor management may yield greater marginal benefits.^25^, ^26^

Our sensitivity analysis showed that the primary conclusions were robust across alternative assumptions. For partial control scenarios, greater population coverage led to higher QALY gains and cost savings, with combined control offering the largest economic benefits. In varying the time to achieve target scenarios, QALYs and cost savings generally declined with slower progress to reach control targets. HbA1c control consistently yielded the highest QALY gains among single risk factor strategies, while combined control was most effective for cost savings. These results highlight the importance of timely and comprehensive risk factor management in the face of real‐world implementation challenges. Varying utility values did not change these trends, except for a potential ceiling effect observed in BP control.

A key strength of our study is the use of population data from the Hospital Authority CMS, a comprehensive data source that allows accurate estimates of the burden related to inadequate risk factor control. By modelling the macrovascular and microvascular outcomes for each individual with type 2 diabetes in Hong Kong, we were able to estimate the long‐term health and economic outcomes of inadequate risk factor control rather than simply cardiovascular risk alone.^27^

Furthermore, our study undertook a comparative analysis of the projected healthcare cost savings associated with individual and combined risk factor control scenarios. Given the chronic nature of diabetes, our modelling framework enabled us to estimate the long‐term health and economic outcomes of different risk factor control strategies. Specifically, we examined the cost savings associated with controlling four individual risk factors (glycaemic, BP, LDL‐cholesterol and BMI) and combined control of risk factors (three risk factors: glycaemic, BP and LDL‐cholesterol). Our findings provide valuable insights into the potential cost benefits of different risk factor control strategies, informing healthcare policymakers and practitioners about the most cost‐saving approaches to reduce the healthcare burden related to type 2 diabetes.

A limitation of the study, common in all health economic modelling analyses, is the uncertainty associated with modelling future outcomes.^28^ We used an updated version of a published health economic outcomes model of type 2 diabetes for Chinese populations that was externally validated against a nationally representative sample of Chinese residents (China Health and Retirement Longitudinal Study [CHARLS] cohort) and nine diabetes trials.^13^ For the updated model, we used data from a population‐based cohort of individuals with type 2 diabetes in Hong Kong Hospital Authority CMS between 2013 and April 2022.^14^ Using a model with the most recent data and keeping parameters (other than HbA1c, BP and cholesterol) constant throughout the time horizon can reduce modelling uncertainty. Another limitation is that these analyses did not account for potential treatment‐related adverse effects, such as hypoglycaemia or hypotension, when aiming for lower risk factor target levels, and these effects should be considered when assessing individualised therapies.^29^ The indirect costs associated with lost workplace productivity were not calculated, as our main study aim was to quantify the direct cost burden associated with diabetes‐related complications arising from inadequate risk factor control. A significant proportion of the study population may not have accrued indirect costs, as the mean age of the diabetes population was beyond the retirement age (set at 60–65 years in Hong Kong) at baseline. Nevertheless, further cost‐effectiveness analyses are required to assess the full economic burden of diabetes to society in Hong Kong.

Our study highlights that combined control of glycaemia, BP and LDL‐cholesterol produces the greatest health and economic benefits for people with type 2 diabetes. The greater cost savings observed in women indicate the potential value of targeted interventions. In Hong Kong, programmes such as Risk Assessment and Management Programme‐Diabetes Mellitus (RAMP‐DM) and the Chronic Disease Co‐Care Pilot Scheme (CDCC) provide strong platforms for comprehensive risk factor management through multidisciplinary care, family doctor models and incentive mechanisms.^30^, ^31^ Such initiatives are essential to drive a shared care model that can translate the potential gains highlighted in our findings into real‐world improvements.

## 5 CONCLUSION

The clinical and economic burden from inadequate control of risk factors in Hong Kong is projected to be substantial. This health and economic burden is potentially reducible and should focus more attention on effective and intensive interventions to meet risk factor targets. Our estimations for healthy QALYs and direct healthcare costs and the findings of the potential for large gains in population health by improved risk factor controls provide a useful health measure and solid evidence for health professionals and policymakers even in high‐income economies with long life expectancies and well‐developed healthcare services.

## AUTHOR CONTRIBUTIONS

JQ conceived the study and designed the study methodology with JC, CSN and YW. AL and YS conducted the analysis and visualised the study results. JQ supervised the study with XX. CLKL, EYFW, and JQ acquired funding. AL, YS and JQ drafted the initial manuscript. All authors interpreted the findings, reviewed and revised the manuscript, and approved the submitted version.

## FUNDING INFORMATION

This work was supported by the Health Bureau, Government of Hong Kong SAR (COVID19F08).

## CONFLICT OF INTEREST STATEMENT

Eric Y. F. Wan has received research grants from the Health Bureau, the Hong Kong Research Grants Council, Narcotics Division, Security Bureau, Social Welfare Department, Labour and Welfare Bureau of the Government of the Hong Kong SAR and National Natural Science Foundation of China; serves as a member of the Core Team for the Expert Group on Drug Registration of the Pharmacy and Poisons Board, and is the director of Advance Data Analytics for Medical Science (ADAMS) Limited (HK). These are outside the submitted work. Cindy L. K. Lam has received research grants from the Health Bureau of the Government of the Hong Kong SAR, the Hong Kong Research Grant Council, the Hong Kong College of Family Physicians and Kerry Group Kuok Foundation, honoraria from the World Organisation of Family Doctors, Malaysian College of Family Physicians (Malaysia) and International Association of Chinese Nephrologist (Hong Kong), College of Family Physicians Singapore, and a conference grant from the Hong Kong College of Family Physicians outside the submitted work. The rest of the authors declare no competing interest. During the preparation of this work the authors used GPT‐4o to improve the language of the work. After using this tool/service, the authors reviewed and edited the content as needed and take full responsibility for the content of the publication.

## PEER REVIEW

The peer review history for this article is available at https://www.webofscience.com/api/gateway/wos/peer-review/10.1111/dom.70081.

## Supporting information


**Data S1.** Supporting Information.

## Data Availability

The data supporting the findings of this study were extracted from the electronic healthcare records of the Hong Kong Hospital Authority. However, due to restrictions on data availability and their use under licence for this study, the data are not publicly accessible. They can, however, be made available by the authors upon reasonable request, subject to approval and compliance with the data access policies of the Hong Kong Hospital Authority.
