# Supplementary material for: The healthcare and economic burden associated with inadequate risk factor control for type 2 diabetes in Hong Kong: A population‐based modelling study
Source: Diabetes Obes Metab. 2025 Sep 10;27(11):6705–14. doi: 10.1111/dom.70081 (PMC12515775; doi:10.1111/dom.70081)
Supplement: Supplementary file 1 — Data S1. Supporting Information. [file DOM-27-6705-s001.docx]

Table of Contents

[Appendix 1. Biomarker progression model specifications 2](#_Toc206306670)

[Table S1. Risk factor targets 3](#_Toc206306675)

[Table S2. Baseline characteristics of study population with type 2 diabetes in 2021 4](#_Toc206306676)

[Table S3. Death rate by year associated with risk factor control (%) 6](#_Toc206306677)

[Table S4. Sensitivity analysis of Quality-adjusted life years (QALYs) associated with risk factor control 7](#_Toc206306678)

[Table S5. Sensitivity analysis of cost savings associated with risk factor control (in million, US$) 10](#_Toc206306679)

[Figure S1. QALY gains of different risk factor control methods in 5 & 10 years by diabetes and 10 related complications 12](#_Toc206306680)

[Figure S2. Cost savings of different risk factor control methods in 5 & 10 years by diabetes and related 12 complications 13](#_Toc206306681)

[Figure S3. Data cleaning process flow chart 14](#_Toc206306682)

[Reference 15](#_Toc206306683)

## Appendix 1. Biomarker progression model specifications

$\boldsymbol{sb}\boldsymbol{p}_{\boldsymbol{j}}\boldsymbol{= 48.3593 + 0.2409 \cdot femal}\boldsymbol{e}_{\boldsymbol{j}}\boldsymbol{+ 0.4164 \cdot sb}\boldsymbol{p}_{\boldsymbol{j-1}}\boldsymbol{+ 0.5707 \cdot lo}\boldsymbol{g} \left( \boldsymbol{duratio}\boldsymbol{n}_{\boldsymbol{j}} \right)\boldsymbol{+ 0.1646 \cdot sb}\boldsymbol{p}_{\boldsymbol{1}}\boldsymbol{+ 0.0820 \cdot ag}\boldsymbol{e}_{\boldsymbol{j}}$ **(1.1)**

$\boldsymbol{bm}\boldsymbol{i}_{\boldsymbol{j}}\boldsymbol{= 6.7280 + 0.0220 \cdot femal}\boldsymbol{e}_{\boldsymbol{j}}\boldsymbol{+ 0.4573 \cdot bm}\boldsymbol{i}_{\boldsymbol{j-1}}\boldsymbol{- 0.0814 \cdot log}\left( \boldsymbol{duratio}\boldsymbol{n}_{\boldsymbol{j}} \right)\boldsymbol{+ 0.3067 \cdot bm}\boldsymbol{i}_{\boldsymbol{1}}\boldsymbol{- 0.0094 \cdot ag}\boldsymbol{e}_{\boldsymbol{j}}$ **(1.2)**

$\boldsymbol{hba}\boldsymbol{1}\boldsymbol{c}_{\boldsymbol{j}}\boldsymbol{= 2.4161 + 0.5294 \cdot hba}\boldsymbol{1}\boldsymbol{c}_{\boldsymbol{j-1}}\boldsymbol{+ 0.0288 \cdot log(duratio}\boldsymbol{n}_{\boldsymbol{j}}\boldsymbol{)+ 0.1437 \cdot hba}\boldsymbol{1}\boldsymbol{c}_{\boldsymbol{1}}\boldsymbol{- 0.0028 \cdot ag}\boldsymbol{e}_{\boldsymbol{j}}$ **(1.3)**

$\boldsymbol{ld}\boldsymbol{l}_{\boldsymbol{j}}\boldsymbol{= 1.0769 + 0.0357 \cdot femal}\boldsymbol{e}_{\boldsymbol{j}}\boldsymbol{+ 0.4777 \cdot ld}\boldsymbol{l}_{\boldsymbol{j-1}}\boldsymbol{- 0.2085 \cdot log(duratio}\boldsymbol{n}_{\boldsymbol{j}}\boldsymbol{)+ 0.1086 \cdot ld}\boldsymbol{l}_{\boldsymbol{1}}\boldsymbol{- 0.0026 \cdot ag}\boldsymbol{e}_{\boldsymbol{j}}\boldsymbol{+ 0.0439 \cdot duratio}\boldsymbol{n}_{\boldsymbol{j}}$ **(1.4)**

Note: j indexes follow-up year (j = 1 is baseline; j − 1 is the immediately preceding year), and the recursions are evaluated for j ≥ 2. sbp_j, bmi_j, hba1c_j, and ldl_j denote systolic blood pressure, body mass index, glycated haemoglobin (HbA1c), and low-density lipoprotein cholesterol at year j, respectively; sbp_1, bmi_1, hba1c_1, and ldl_1 are their baseline values. female_j is a sex indicator (1 = female, 0 = male; treated as time-invariant in our data), age_j is age in years at year j, and duration_j is time since diagnosis at year j; log(·) denotes the natural logarithm. The individual index is suppressed for clarity—the equations are specified within person over time. A linear duration term is included only in the LDL equation. Units follow those recorded in the dataset.

## Table S1. Risk factor targets

|  | Health Bureau^1^ | ADA^2^ | NICE^3,4^ | MOH^5^ | CDS^6^ |
| --- | --- | --- | --- | --- | --- |
| HbA1c | < 7% (53 mmol/mol) | < 7% (53 mmol/mol) | < 6.5% (48 mmol/mol) | < 7% (53 mmol/mol) | < 7% (53 mmol/mol) |
| LDL | < 2.6 mmol/L;  < 1.8 mmol/L with pre-existing CVD | < 1.83 mmol/L  (70 mg/dL) | N/A | < 2.6 mmol/L;  < 2.1 mmol/L with overt CVD/  CKD | < 2.6 mmol/L;  < 1.8 mmol/L with ASCVD |
| BP | < 130/80 mmHg | < 130/80 mmHg;  < 140/90 mmHg for individuals with 10-year ASCVD risk <15% | < 140/90 mmHg for age under 80;  < 150/90 mmHg for aged 80 and over | < 140/80 mmHg | < 130/80 mmHg |
| BMI | < 25 kg/m^2^ | <25 kg/m^2^;  <23 kg/ m^2^ for Asian American | N/A | <25 kg/m^2^ | <24kg/m^2^ |

Abbreviations: NICE, National Institute for Health and Care Excellence; ADA, American Diabetes Association; MOH, Ministry of Health; CDS, Chinese Diabetes Society; HbA1c, glycated haemoglobin; BP, blood pressure; LDL, LDL-cholesterol; CVD, cardiovascular disease; CKD, chronic kidney disease; ASCVD, atherosclerotic cardiovascular disease.

## Table S2. Baseline characteristics of study population with type 2 diabetes in 2021

|  | **Mean (SD)** | | | **Median (SD)** | | |
| --- | --- | --- | --- | --- | --- | --- |
| **Characteristic** | **Male** | **Female** | **Total** | **Male** | **Female** | **Total** |
| N | 262,018 | 264,654 | 526,672 | 262,018 | 264,654 | 526,672 |
| Female (%) | 0 | 100 | 50·3 | 0 | 100 | 50·3 |
| Age (years) | 67·0 (11·9) | 69·4 (12·6) | 68·2 (12·3) | 67·0 (11·9) | 69·0 (12·6) | 68·0 (12·3) |
| Duration of diabetes (years) | 6·4 (4·0) | 6·9 (4·0) | 6·7 (4·0) | 7·0 (4·0) | 8·0 (4·0) | 7·0 (4·0) |
| HbA1c (%) | 7·2 (1·3) | 7·1 (1·1) | 7·1 (1·2) | 6·9 (1·3) | 6·9 (1·1) | 6·9 (1·2) |
| Systolic blood pressure (mmHg) | 134·3 (15·5) | 135·2 (16·2) | 134·8 (15·9) | 135·3 (15·5) | 135·3 (16·2) | 135·3 (15·9) |
| Diastolic blood pressure (mmHg) | 73·9 (10·5) | 70·8 (10·4) | 72·3 (10·5) | 71·8 (10·5) | 69·6 (10·4) | 70·0 (10·5) |
| LDL cholesterol (mmol/L) | 2·1 (0·7) | 2·2 (0·7) | 2·1 (0·7) | 2·1 (0·7) | 2·1 (0·7) | 2·1 (0·7) |
| HDL cholesterol (mmol/L) | 1·2 (0·3) | 1·4 (0·3) | 1·3 (0·3) | 1·2 (0·3) | 1·4 (0·3) | 1·3 (0·3) |
| BMI (kg/m^2^) | 25·8 (3·3) | 25·7 (3·6) | 25·8 (3·4) | 25·8 (3·3) | 25·8 (3·6) | 25·8 (3·4) |
| Smokers (%) | 6·8 | 0·9 | 3·8 | 6·8 | 0·9 | 3·8 |
| Ex-smokers (%) | 11·3 | 1·1 | 6·2 | 11·3 | 1·1 | 6·2 |
| Triglycerides (mmol/L) | 1·5 (1·1) | 1·5 (1·0) | 1·5 (1·0) | 1·3 (1·1) | 1·4 (1·0) | 1·4 (1·0) |
| Estimated glomerular filtration rate (mL/min/1.73m^2^) | 74·1 (23·0) | 75·5 (23·6) | 74·8 (23·3) | 74·2 (23·1) | 75·5 (23·8) | 74·9 (23·5) |
| Haemoglobin (g/dL) | 12·9 (1·6) | 11·9 (1·3) | 12·4 (1·5) | 12·5 (1·6) | 11·5 (1·3) | 12·5 (1·5) |
| Disease History (%) | | | | | | |
| Amputation | 0·05 | 0·0004 | 0·024 | 0·05 | 0·0004 | 0·024 |
| Cataract | 11·7 | 15·3 | 13·5 | 11·7 | 15·3 | 13·5 |
| Ischemic heart disease | 13·4 | 8·4 | 10·9 | 13·4 | 8·4 | 10·9 |
| Heart failure | 3·7 | 3·9 | 3·8 | 3·7 | 3·9 | 3·8 |
| Renal failure | 5·2 | 3·7 | 4·4 | 5·2 | 3·7 | 4·4 |
| Neuropathy | 2·1 | 0·9 | 1·5 | 2·1 | 0·9 | 1·5 |
| Retinopathy | 8·5 | 7·5 | 8·0 | 8·5 | 7·5 | 8·0 |
| Ulcer skin | 1·6 | 1·3 | 1·4 | 1·6 | 1·3 | 1·4 |
| Myocardial infarction | 3·9 | 2·0 | 2·9 | 3·9 | 2·0 | 2·9 |
| Stroke | 3·1 | 2·9 | 3·0 | 3·1 | 2·9 | 3·0 |
| Atrial fibrillation | 4·4 | 4·0 | 4·2 | 4·4 | 4·0 | 4·2 |
| Peripheral vascular disease | 1·4 | 0·8 | 1·1 | 1·4 | 0·8 | 1·1 |
| Haemodialysis | 0·92 | 0·54 | 0·73 | 0·92 | 0·54 | 0·73 |

Abbreviations: HbA1c, glycated haemoglobin; BMI, body mass index.

## Table S3. Death rate by year associated with risk factor control (%)

| **Year** | **HbA1c** | | **Blood Pressure** | | **LDL-cholesterol** | | **Combined Control** | |
| --- | --- | --- | --- | --- | --- | --- | --- | --- |
|  | **baseline** | **target** | **baseline** | **target** | **baseline** | **target** | **baseline** | **target** |
| 1 | 1·45 | 1·98 | 1·90 | 2·02 | 1·10 | 1·05 | 1·72 | 2·10 |
| 2 | 2·04 | 2·51 | 2·44 | 2·48 | 1·54 | 1·42 | 2·29 | 2·51 |
| 3 | 2·53 | 2·68 | 2·82 | 2·84 | 1·72 | 1·78 | 2·68 | 2·83 |
| 4 | 2·94 | 2·99 | 3·14 | 3·13 | 2·04 | 2·15 | 3·00 | 2·98 |
| 5 | 3·23 | 3·18 | 3·46 | 3·37 | 2·33 | 2·34 | 3·30 | 3·28 |
| 6 | 3·54 | 3·47 | 3·74 | 3·70 | 2·45 | 2·51 | 3·58 | 3·52 |
| 7 | 3·87 | 3·76 | 3·95 | 4·00 | 2·82 | 2·78 | 3·86 | 3·77 |
| 8 | 4·13 | 4·05 | 4·30 | 4·33 | 3·07 | 3·08 | 4·19 | 4·01 |
| 9 | 4·45 | 4·23 | 4·58 | 4·58 | 3·32 | 3·35 | 4·46 | 4·38 |
| 10 | 4·72 | 4·66 | 4·88 | 4·85 | 3·51 | 3·48 | 4·76 | 4·62 |

Abbreviations: HbA1c, solely control of glycated haemoglobin; Blood Pressure, solely control of blood pressure; LDL-cholesterol, solely control of LDL-cholesterol; Combined Control, combined control of HbA1c, blood pressure, and LDL-cholesterol; baseline, reference scenario that has no control on any risk factor; target, target scenario that control corresponding risk factor(s).

## Table S4. Sensitivity analysis of Quality-adjusted life years (QALYs) associated with risk factor control

|  | **All** | | **Male** | | **Female** | |
| --- | --- | --- | --- | --- | --- | --- |
|  | **5-year** | **10-year** | **5-year** | **10-year** | **5-year** | **10-year** |
| **Partial control of target population** | | | | | | |
| HbA1c | | | | | | |
| 50% | 8,697 | 15,517 | 5,657 | 9,689 | 3,017 | 5,832 |
| 75% | 13,046 | 22,756 | 8,351 | 14,451 | 4,809 | 8,379 |
| 100% | 17,156 | 29,699 | 10,705 | 18,650 | 6,370 | 10,962 |
| Blood Pressure | | | | | | |
| 50% | 632 | 1,780 | 1,778 | 3,203 | -1,145 | -1,423 |
| 75% | 735 | 3,123 | 959 | 2,129 | -225 | 993 |
| 100% | 1,756 | 3,033 | 767 | 670 | 989 | 2,362 |
| LDL-cholesterol | | | | | | |
| 50% | 121 | -41 | 242 | 212 | -312 | -676 |
| 75% | 374 | 721 | 396 | 629 | -139 | -302 |
| 100% | 543 | 842 | 417 | 741 | 42 | 112 |
| HbA1c + BP + LDL | | | | | | |
| 50% | 8,925 | 15,414 | 5,830 | 10,003 | 3,082 | 5,636 |
| 75% | 12,846 | 22,416 | 8,200 | 14,238 | 4,454 | 8,255 |
| 100% | 16,758 | 29,138 | 10,655 | 18,312 | 6,132 | 11,241 |
| **Time varying to target** | | | | | | |
| HbA1c | | | | | | |
| 1-year | 17,156 | 29,699 | 10,705 | 18,650 | 6,370 | 10,962 |
| 3-year | 10,055 | 19,332 | 6,534 | 12,305 | 3,531 | 6,749 |
| 5-year | 7,286 | 17,823 | 4,942 | 11,685 | 2,412 | 6,386 |
| Blood Pressure | | | | | | |
| 1-year | 1,756 | 3,033 | 767 | 670 | 989 | 2,362 |
| 3-year | 1,545 | 2,569 | 1,274 | 1,667 | 482 | 1,414 |
| 5-year | 907 | 2,011 | 968 | 1,625 | -167 | 665 |
| LDL-cholesterol | | | | | | |
| 1-year | 543 | 842 | 417 | 741 | 42 | 112 |
| 3-year | 479 | 358 | 360 | 392 | 127 | -36 |
| 5-year | 476 | 112 | 565 | 605 | -29 | -396 |
| HbA1c + BP + LDL | | | | | | |
| 1-year | 16,758 | 29,138 | 10,655 | 18,312 | 6,132 | 11,241 |
| 3-year | 6,436 | 5,574 | 4,082 | 4,111 | 2,020 | 1,110 |
| 5-year | 6,768 | 7,571 | 4,574 | 5,471 | 1,999 | 1,779 |
| **Utility value variation** | | | | | | |
| HbA1c | | | | | | |
| lower | 15,391 | 26,900 | 9,551 | 16,799 | 5,780 | 10,081 |
| normal | 17,156 | 29,699 | 10,705 | 18,650 | 6,370 | 10,962 |
| upper | 17,267 | 29,868 | 10,768 | 18,756 | 6,418 | 10,987 |
| Blood Pressure | | | | | | |
| lower | 1,486 | 2,528 | 562 | 386 | 924 | 2,142 |
| normal | 1,756 | 3,033 | 767 | 670 | 989 | 2,362 |
| upper | 1,658 | 2,875 | 733 | 603 | 925 | 2,273 |
| LDL-cholesterol | | | | | | |
| lower | 449 | 741 | 336 | 642 | 33 | 124 |
| normal | 543 | 842 | 417 | 741 | 42 | 112 |
| upper | 452 | 747 | 381 | 674 | 9 | 63 |
| HbA1c + BP + LDL | | | | | | |
| lower | 15,136 | 26,645 | 9,539 | 16,539 | 5,583 | 10,452 |
| normal | 16,758 | 29,138 | 10,655 | 18,312 | 6,132 | 11,241 |
| upper | 16,695 | 28,883 | 10,693 | 18,276 | 6,027 | 11,004 |

Note: Table S4 presents the results of sensitivity analysis of quality-adjusted life years (QALYs) under different risk factor control strategies. Median values for each group (all population, male, female) were calculated from 20 independent simulations and are presented in the table.

Three sensitivity conditions were tested:
(1) Partial control of the target population, with scenarios where 50%, 75%, or 100% of the population achieve the risk factor control targets;
(2) Time varying to target, with scenarios where targets are reached in 1, 3, or 5 years;
(3) Utility value variation, with scenarios applying lower, normal, or higher sets of utility values for complications.

Abbreviations: HbA1c, solely control of glycated haemoglobin; Blood Pressure, solely control of blood pressure; LDL-cholesterol, solely control of LDL-cholesterol; HbA1c + BP + LDL, combined control of HbA1c, blood pressure, and LDL-cholesterol; 50%, 75%, 100% (partial control of target population): proportion of the population achieving the risk factor target; 1-year, 3-year, 5-year (time varying to target): duration required to achieve the risk factor control target; lower, normal, upper (utility value variation): lower, normal, or higher sets of utility values applied in the calculation.

## Table S5. Sensitivity analysis of cost savings associated with risk factor control (in million, US$)

|  | **All** | | **Male** | | **Female** | |
| --- | --- | --- | --- | --- | --- | --- |
|  | **5-year** | **10-year** | **5-year** | **10-year** | **5-year** | **10-year** |
| **Partial control of target population** | | | | | | |
| HbA1c | | | | | | |
| 50% | 0.1 | 18.5 | -3.8 | 12.5 | 2.8 | 5.5 |
| 75% | 0.3 | 18.6 | -5.4 | 7.4 | 5.0 | 14.4 |
| 100% | 5.2 | 36.3 | 1.1 | 12.6 | 4.0 | 18.1 |
| Blood Pressure | | | | | | |
| 50% | 6.7 | 18.0 | 2.8 | 7.9 | 3.9 | 10.0 |
| 75% | 10.1 | 25.9 | 4.5 | 11.3 | 5.7 | 14.8 |
| 100% | 13.8 | 35.5 | 6.0 | 15.6 | 7.6 | 19.9 |
| LDL-cholesterol | | | | | | |
| 50% | 1.9 | 10.3 | 0.3 | 2.9 | 2.3 | 7.1 |
| 75% | 2.4 | 8.9 | -0.3 | 4.3 | 3.5 | 5.2 |
| 100% | 3.7 | 12.5 | 0.6 | 7.3 | 2.1 | 2.7 |
| HbA1c + BP + LDL | | | | | | |
| 50% | 21.5 | 77.0 | 5.4 | 25.0 | 15.9 | 44.4 |
| 75% | 24.4 | 97.8 | 4.0 | 35.3 | 21.5 | 64.9 |
| 100% | 34.2 | 121.0 | 6.8 | 43.9 | 24.7 | 77.3 |
| **Time varying to target** | | | | | | |
| HbA1c | | | | | | |
| 1-year | 5.2 | 36.3 | 1.1 | 12.6 | 4.0 | 18.1 |
| 3-year | 24.2 | 76.8 | 10.5 | 46.3 | 13.7 | 32.6 |
| 5-year | 12.3 | 42.3 | 4.2 | 26.6 | 6.5 | 16.4 |
| Blood Pressure | | | | | | |
| 1-year | 13.8 | 35.5 | 6.0 | 15.6 | 7.6 | 19.9 |
| 3-year | 11.0 | 41.6 | -6.0 | -2.7 | 16.1 | 42.6 |
| 5-year | 4.0 | 38.8 | -6.5 | -2.0 | 10.6 | 36.0 |
| LDL-cholesterol | | | | | | |
| 1-year | 3.7 | 12.5 | 0.6 | 7.3 | 2.1 | 2.7 |
| 3-year | -0.7 | -2.0 | 0.0 | 2.8 | 1.1 | -2.7 |
| 5-year | -1.8 | -0.2 | -2.1 | -0.6 | 0.9 | 0.1 |
| HbA1c + BP + LDL | | | | | | |
| 1-year | 34.2 | 121.0 | 6.8 | 43.9 | 24.7 | 77.3 |
| 3-year | 0.2 | 25.6 | 2.7 | 22.5 | 1.1 | -0.5 |
| 5-year | -4.5 | -16.0 | -14.7 | -16.2 | 9.2 | 2.0 |

Note: Table S5 presents the results of sensitivity analysis of cost savings under different risk factor control strategies. Median values for each group (all population, male, female) were calculated from 20 independent simulations and are presented in the table.

Two sensitivity conditions were tested:
(1) Partial control of the target population, with scenarios where 50%, 75%, or 100% of the population achieve the risk factor control targets;
(2) Time varying to target, with scenarios where targets are reached in 1, 3, or 5 years.

All results in Table S5 are presented in million USD.

Abbreviations: HbA1c, solely control of glycated haemoglobin; Blood Pressure, solely control of blood pressure; LDL-cholesterol, solely control of LDL-cholesterol; HbA1c + BP + LDL, combined control of HbA1c, blood pressure, and LDL-cholesterol; 50%, 75%, 100% (partial control of target population): proportion of the population achieving the risk factor target; 1-year, 3-year, 5-year (time varying to target): duration required to achieve the risk factor control target.

## Figure S1. QALY gains of different risk factor control methods in 5 & 10 years by diabetes related complications


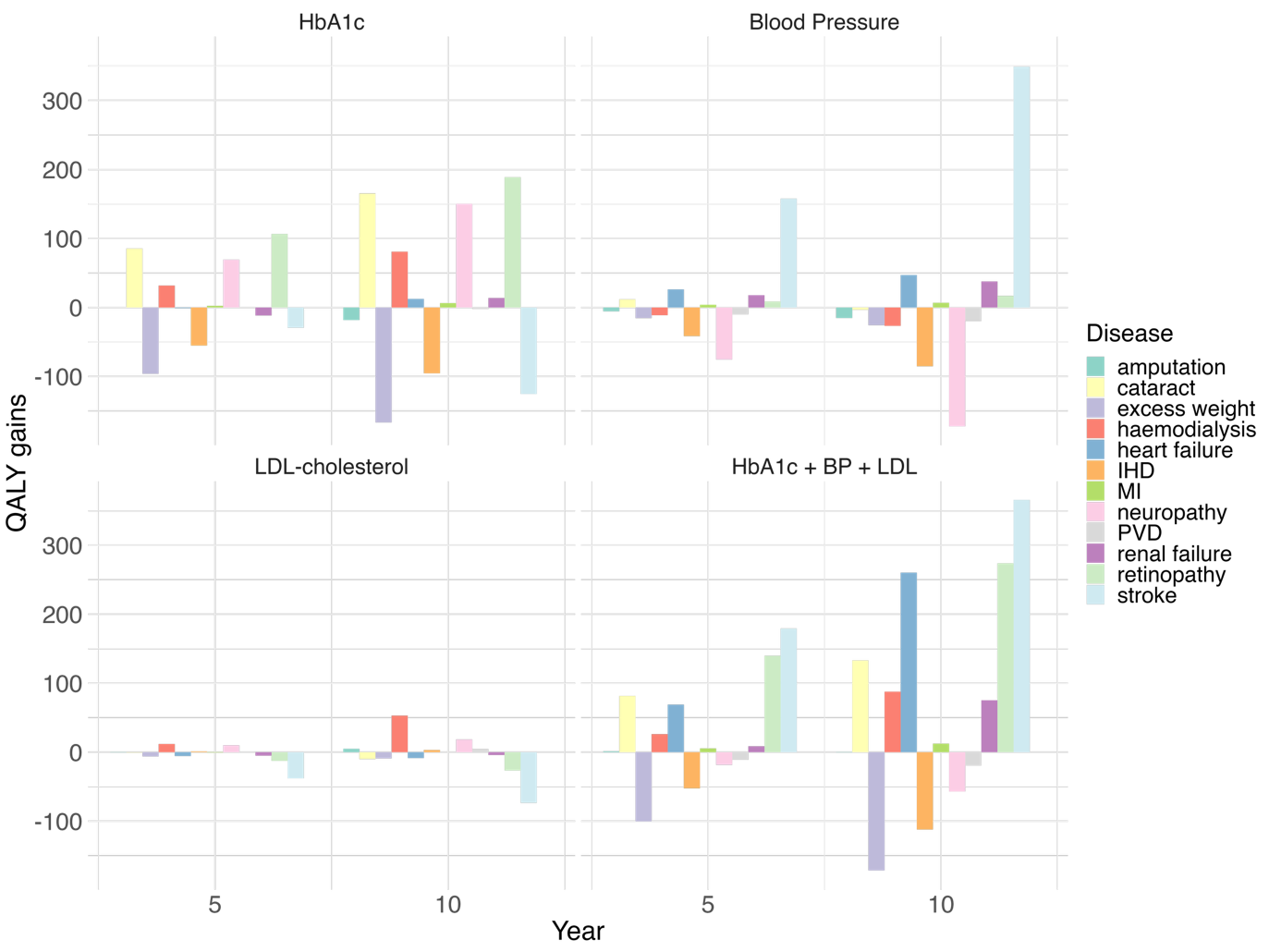


Abbreviations: QALY, quality-adjusted life years; HbA1c, solely control of glycated haemoglobin; Blood Pressure, solely control of blood pressure; LDL-cholesterol, solely control of LDL-cholesterol; HbA1c + BP + LDL, combined control of HbA1c, blood pressure, and LDL-cholesterol; excess weight, MI, myocardial infarction; IHD, ischaemic heart disease; PVD, peripheral vascular disease.

Note: Negative QALY gains indicate a decrease in QALYs compared to the reference scenario.

## Figure S2. Cost savings of different risk factor control methods in 5 & 10 years by diabetes and related 12 complications

**
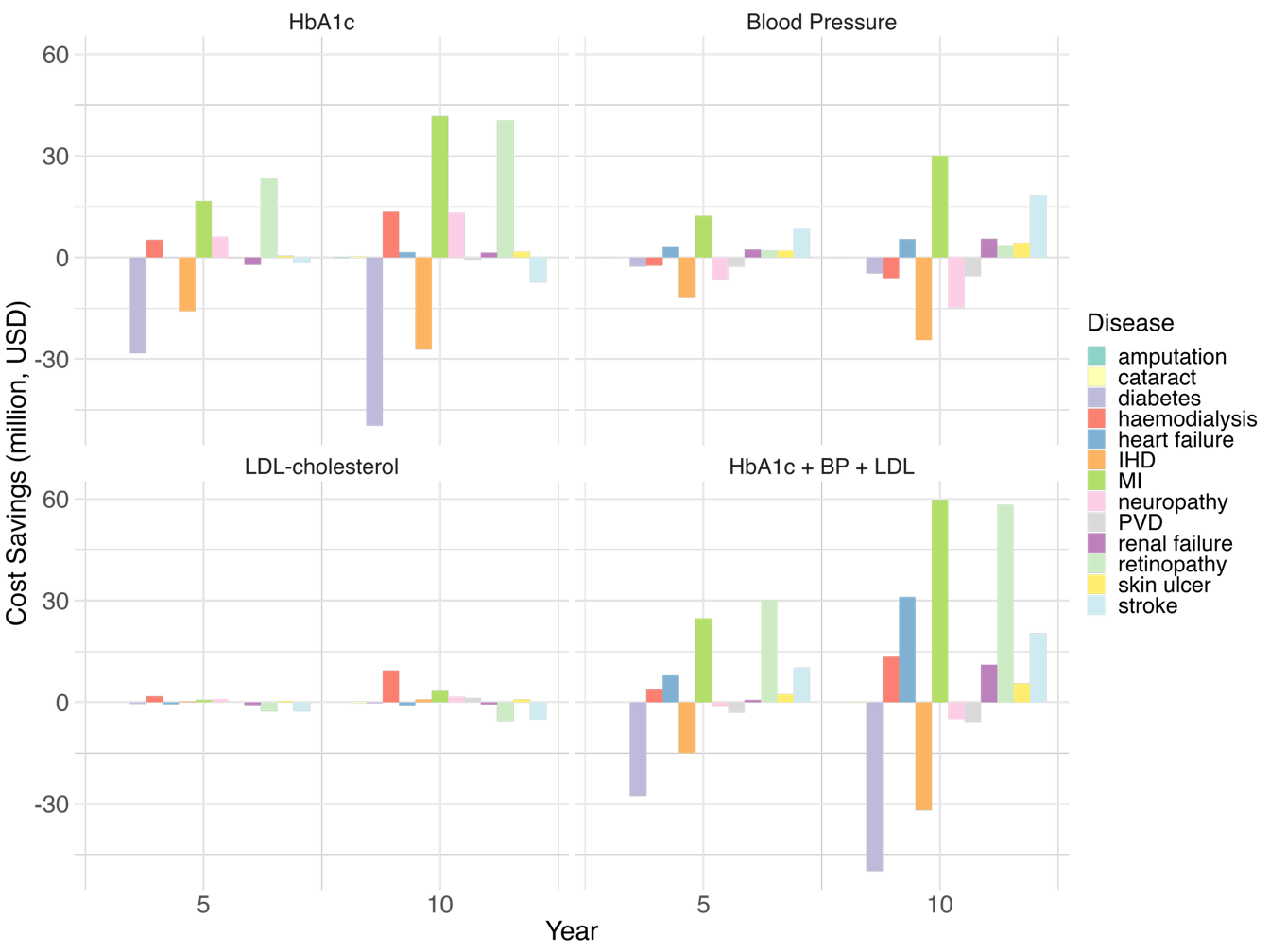
**

Abbreviations: HbA1c, solely control of glycated haemoglobin; Blood Pressure, solely control of blood pressure; LDL-cholesterol, solely control of LDL-cholesterol; HbA1c + BP + LDL, combined control of HbA1c, blood pressure, and LDL-cholesterol; MI, myocardial infarction; IHD, ischaemic heart disease; PVD, peripheral vascular disease.

Note: Negative cost savings indicate an increase in costs compared to the reference scenario.

## Figure S3. Data cleaning process flow chart


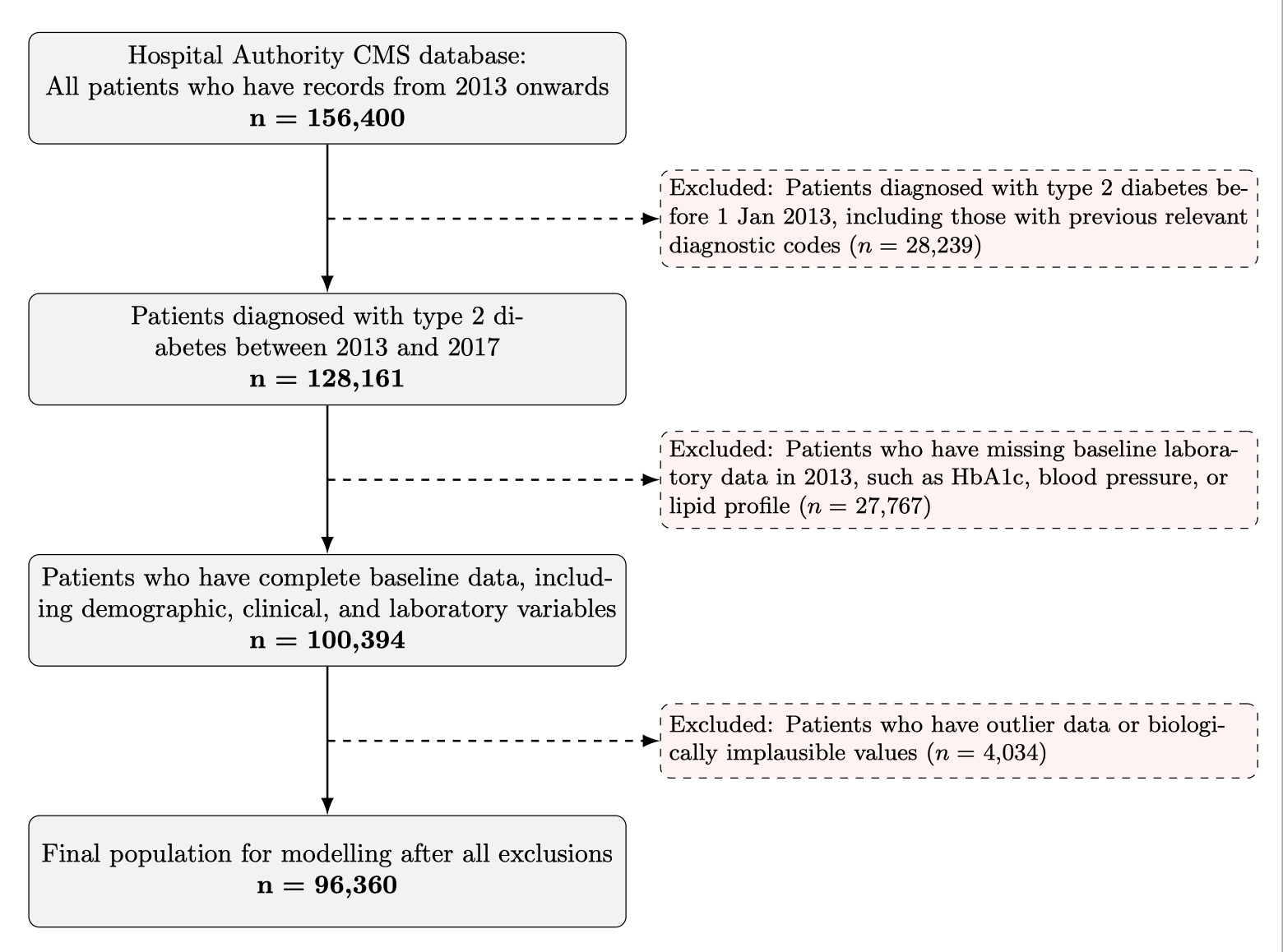


Note: This flowchart illustrates the process of study population selection from the Hospital Authority Clinical Management System (CMS). Patients were included if they were diagnosed with type 2 diabetes between 2013 and 2017 and had complete baseline data. Exclusion criteria comprised a prior diagnosis of type 2 diabetes before 2013, missing baseline laboratory data in 2013, and biologically implausible outlier data. The final analytic cohort consisted of 96,360 eligible participants.

## Reference

1. Diabetes Care [Internet]. [cited 2025 Jan 14]. Available from: https://www.healthbureau.gov.hk/phcc/rfs/english/reference_framework/diabetes_care.html

2. Fox CS, Golden SH, Anderson C, Bray GA, Burke LE, de Boer IH, et al. Update on Prevention of Cardiovascular Disease in Adults With Type 2 Diabetes Mellitus in Light of Recent Evidence: A Scientific Statement From the American Heart Association and the American Diabetes Association. Circulation. 2015 Aug 25;132(8):691–718.

3. Overview | Type 2 diabetes in adults: management | Guidance | NICE [Internet]. [cited 2025 Apr 26]. Available from: https://www.nice.org.uk/guidance/ng28

4. Overview | Hypertension in adults: diagnosis and management | Guidance | NICE [Internet]. NICE; 2019 [cited 2025 Apr 26]. Available from: https://www.nice.org.uk/guidance/ng136

5. Goh SY, Ang SB, Bee YM, Chen RY, Gardner D, Ho E, et al. Ministry of Health Clinical Practice Guidelines: Diabetes Mellitus. Singapore Med J. 2014 Jun;55(6):334–47.

6. Jia W, Weng J, Zhu D, Ji L, Lu J, Zhou Z, et al. Standards of medical care for type 2 diabetes in China 2019. Diabetes Metab Res Rev. 2019 Sep;35(6):e3158.
